# Pulmonary Hypertension in Parenchymal Lung Disease

**DOI:** 10.1155/2012/684781

**Published:** 2012-10-10

**Authors:** Iraklis Tsangaris, Georgios Tsaknis, Anastasia Anthi, Stylianos E. Orfanos

**Affiliations:** 2nd Department of Critical Care and Pulmonary Hypertension Clinic, Attikon University Hospital, University of Athens Medical School, 1 Rimini Street, Haidari, 12462 Athens, Greece

## Abstract

Idiopathic pulmonary arterial hypertension (IPAH) has been extensively investigated, although it represents a less common form of the pulmonary hypertension (PH) family, as shown by international registries. Interestingly, in types of PH that are encountered in parenchymal lung diseases such as interstitial lung diseases (ILDs), chronic obstructive pulmonary disease (COPD), and many other diffuse parenchymal lung diseases, some of which are very common, the available data is limited. In this paper, we try to browse in the latest available data regarding the occurrence, pathogenesis, and treatment of PH in chronic parenchymal lung diseases.

## 1. Introduction

Pulmonary arterial hypertension (PAH) is defined as mean pulmonary artery pressure (mPAP) ≥25 mmHg at rest, with a mean pulmonary capillary wedge pressure (PCWP), left atrial pressure or left ventricular end-diastolic pressure (LVEDP) less than or equal to 15 mmHg validated by right heart catheterization (RHC) [1]. These values are being used by all PAH registries and in all randomized controlled trials (RCTs) [2–7]. Pressure measurements during exercise are no longer recommended or supported by data for pulmonary hypertension (PH) diagnosis. As of 2009, based on the latest Dana Point Classification [1], PH due to underlying parenchymal diseases, such as COPD and interstitial lung disease (ILD), remains in group 3. Other diseases with multisystemic, and more importantly pulmonary, manifestations such as connective tissue diseases (CTDs), or sarcoidosis are categorized separately (groups 1.4.1 and 5.2, resp.). 

In patients with parenchymal lung disease, PH is reported likely modest (mPAP = 25 to 35 mmHg), although in some subjects PAP can be markedly increased (mPAP = 35 to 50 mmHg) [8, 9]. In such patients, especially in those who have mild-to-moderate impaired lung mechanics, this pressure increase is considered as “out-of-proportion” PH. As an example, in a retrospective study regarding RHC measurements in COPD patients, moderate-to-severe PH (mPAP > 40 mmHg) has been found in only 1% of the study population [9].

Recently, the German consensus group attempted to define severe PH in patients with chronic lung disease according to the following criteria (at least two out of three have to meet): (a) mPAP > 35 mmHg, (b) mPAP ≥ 25 mmHg with limited cardiac index (CI < 2.0 L/min/m^2^), and (c) PVR > 480 dyn/s/cm^−5^ [10]. This definition describes less than 5% of patients with lung disease and gives a quantitative dimension to the “out-of-proportion” approach.

**Figure 1:**
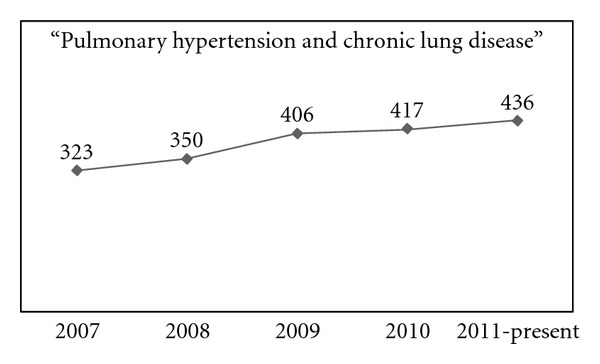
Distribution of PubMed search results within the last 5 years, per calendar year, with the search terms “Pulmonary hypertension and chronic lung disease.” Results contain original research articles, experimental articles, reviews, and case reports.

Epidemiological input on the prevalence of “out-of-proportion” PH is not available, except for few scattered data from subgroup analyses out of large studies. In a survey by a cardiac echo laboratory, the prevalence of all-cause PH (determined as systolic PAP > 40 mmHg) was 10.5% [11]. Among those subjects, only 9.7% had underlying lung diseases and hypoxia. In general, there is limited, albeit adding up data (Figure 1) regarding “out-of-proportion” PH due to chronic lung disease.

## 2. Pathophysiology

The pathophysiological mechanism in “out-of-proportion” PH due to parenchymal lung disease is multifactorial and depending on the underlying type of lung parenchymal involvement. In general, mechanisms include chronic hypoxic vasoconstriction (which is a major factor), mechanical lung stress, capillary loss, smoking habit effects, and inflammation. The acute hypoxic effect in systemic circulation is vasodilation, whereas in pulmonary circulation it triggers an acute vasoconstrictive process regulated by endothelin, serotonin, and other compounds and mediated by ion-channel activity in pulmonary arterial smooth muscle cells (SMCs) [12]. Additively, pulmonary vascular endothelial cells appear to exhibit a paracrine-like activity, metabolizing and uptaking vasoactive compounds that act on the pulmonary vascular tone under hypoxic state, probably contributing to hypoxic vasoconstriction [13].

On the other hand, in chronic hypoxia, which is the case in parenchymal lung disease, it seems that multiple pathological changes may occur in pulmonary vasculature, such as fibrous remodeling and an increase in both the number and mass of SMCs in the arterial wall, resulting in higher PVR over time and development of PH [14, 15]. In animal models, acute and chronic hypoxia appears to share causative intercessors in the disease cascade [16]; therefore, hypoxia may not only start the PH process but also encumber the disease, if not reversed.

In idiopathic pulmonary arterial hypertension (IPAH) and other forms of PH, it is widely accepted that a key histological finding is the plexiform lesion seen in the vast majority of patients [17, 18]. The plexiform lesion develops when capillary formations produce a network that spans in the lumens of dilated thin-walled, small pulmonary arteries. Medial hypertrophy also can be present in smaller arterioles, caused by intimal thickening attributable to the accumulation of one or more layers of myofibroblasts and fibrous matrix proteins within the neointimal space between the endothelium (tunica intima) and the internal elastic lamina. In more advanced stages, small pre- and intra-acinar arterioles predominantly exhibit complex lesions, that cause occlusion of the vessel's lumen, including concentric laminar intimal proliferation, called “onion skin” or concentric-obliterative lesions, and glomeruloid-like plexiform lesions [19]. Interestingly, this lesion was found to be similar in histological appearance with those occurring in limited cutaneous systemic sclerosis (lc-SSc) [20]. However, almost a decade later, it was reported that lc-SSc lesions were all polyclonal, in contrast with plexiform lesions in IPAH which were mainly monoclonal (80%) [21].

## 3. Diagnostic Approach

Due to the limitations of the invasive, albeit consistent, and accurate RHC, transthoracic tissue Doppler echocardiography (TTE) has emerged to fill the diagnostic gap and noninvasively assess the systolic pulmonary artery pressure (sPAP) in order to detect PH at an earlier stage. This technique, when applied by well-trained experts, can be very useful as a “sentinel” study prior to RHC. On the other hand, there is a possibility of misinterpretation which may lead to PH misdiagnosis and devastating consequences [38]. It should be emphasized that TTE can provide only estimates of pulmonary arterial pressures and that RHC is needed in order to establish PH diagnosis. The technique of TTE has been used widely not only as the study of choice in PH screening, but also as the “gold standard” frequent follow-up study in patients under treatment [39]. In a recent prospective trial assessing TTE and RHC measurements in 155 PH patients, there was a significant correlation between RHC and TTE [40]. More specifically, single TTE parameters performed well in predicting final PH diagnosis in this cohort, such as sPAP (area under the curve (AUC) 0.63, *P* = 0.025), the lateral apical RV longitudinal strain (RVaSl) (AUC 0.76, *P* = 0.001), and the ratio of transmitral Doppler early filling velocity to tissue Doppler early diastolic mitral annular velocity (E/E′) (AUC 0.84, *P* < 0.001). In addition, TTE showed a sensitivity of 33.33% and specificity of 100% in all-type precapillary PH identification, as well as 84.72% negative predictive value (NPV) to rule out the disease. However, in a recent analysis of subjects from the REVEAL (Registry to Evaluate Early and Long-term PAH Disease Management) study by Farber et al. [41], in 1883 patients that underwent both RHC and TTE, with the reservation that there were cases where several months have passed in between the tests, there was little association between serial TTE and RHC values. Additionally, repeat TTE measurements alone have shown to be insufficient to accurately monitor changes in PAP or disease progression.

Nowadays, TTE remains unable to replace RHC in establishing PH diagnosis, although it is very reliable for screening, following up, and providing indices of disease severity [42]; furthermore, TTE may distinguish pre- from postcapillary PH in certain cases.

## 4. Pulmonary Arterial Hypertension Associated with Connective Tissue Diseases That Affect Lung Parenchyma 

Pulmonary hypertension is an increasingly recognizable complication and a major cause of death in patients with connective tissue diseases (CTDs), notably occurring in systemic sclerosis (SSc), systemic lupus erythematosus (SLE), rheumatoid arthritis (RA), and mixed CTD (MCTD), overall affecting 3% to 13% of such patients [43–46]; these pathologies may develop solely or in association with ILD [47, 48].

Originally, pulmonary hypertension in IPAH and CTD was thought to share similar histologic and pathophysiologic characteristics [49, 50]. However, there is growing clinical evidence regarding differences in the disease process between CTD-PH and IPAH, in terms of endothelial and metabolic functions, as well as histological trails [51, 52]. In a study regarding the expression and activity of pulmonary endothelial angiotensin-converting enzyme (ACE), endothelial metabolic dysfunction was noticed in CTD-PH, compared to a group of IPAH patients. There was also functional evidence that a reduced DLCO value in patients with PAH-CTD was related to the degree of functional capillary surface area (FCSA) loss [53]. It is of interest that pulmonary endothelial dysfunction, based on the aforementioned techniques, was seen in patients with limited and diffuse systemic sclerosis at early disease stages, prior to PH development [54]. These findings could at least partly justify the worse prognosis in such patients, despite their hemodynamic semblance with IPAH [55, 56].

Beside direct involvement of pulmonary vasculature (i.e., group 1), pulmonary hypertension in CTDs may be due to left heart disease, lung parenchyma involvement, chronic thromboembolism (related to groups 2, 3, and 4 resp.), or even venoocclusive disease, often presenting a difficult diagnostic challenge.

### 4.1. Polymyositis/Dermatomyositis and Pulmonary Hypertension

These myopathies are part of the idiopathic inflammatory myopathies family, characterized by proximal muscle weakness, elevated serum creatine kinase, abnormal appearance in electromyography, and inflammatory cell infiltration in muscles. In polymyositis and dermatomyositis (PM/DM), involvement of multiple organs is common [57–59]. The most common affected site, apart from muscles, is the lung, with the general pulmonary complications reaching 40% in such patients, resulting in significantly high mortality rates [60].

Pathogenesis is incompletely understood, with the obvious factor being the autoimmunity as PM/DM commonly presents along with other autoimmune diseases. Recent data suggest a genetic base of the disease that might predispose to autoimmunity [61–63]. In PM specifically, the muscle fiber seems to be the main target. On the other hand, DM is characterized by deposition of membrane attack complex in muscle capillaries. Interestingly, antinuclear and anticytoplasmic autoantibodies are found in up to 90% of patients with PM and DM, allowing clinicians to define homogenous cohorts of PM/DM patients [64].

Pulmonary hypertension occurrence in PM/DM is not thoroughly designated, with available data only in a case report basis. The majority of patients present with breathlessness in effort and pulmonary function test restriction or DLCO decrease. It is of interest that PH in PM/DM affects mainly females [65, 66]. In one autopsy series, 20% of patients with PM had pulmonary arterial medial and intimal hypertrophy, a clue that could be linked to PH pathogenesis in such patients [67]. Another major factor in the PM/DM-PH pathogenesis could be the presence of ILD, that is quite common in the disease (5% to 65%) [68, 69]. True prevalence of PM/DM-PH is still not known, underlining the need for earlier referral of patients and RHC diagnosis confirmation.

### 4.2. Systemic Sclerosis and Pulmonary Hypertension

Systemic sclerosis (SSc) is a chronic systemic autoimmune disease characterized by fibrosis, vascular alterations, and autoantibodies. It is mainly expressed in two forms, (i) the limited systemic sclerosis-scleroderma with cutaneous manifestations such as CREST (calcinosis, Raynaud's phenomenon, esophageal dysmotility, sclerodactyly, telangiectasias) syndrome, a term recently quite abandoned, and (ii) the diffuse systemic sclerosis-scleroderma, which is rapidly progressive and is characterized by multiple internal organ involvement usually including interstitial lung disease of progressive severity [70].

The prevalence of PH in patients suffering from SSc is reported to be 7% to 35%, depending on the cohort studied [46, 71, 72]. Unfortunately, at the time of SSc-PH diagnosis, the plurality of these patients has been reported to be already in New York Heart Association (NYHA) functional class (FC) III or IV, which is of poorer survival compared to NYHA-FC II patients [1, 73–75].

Interestingly, in recent data reported by the French PAH-SSc Network [76], a considerable number of patients in NYHA-FC II with mild symptoms at the time of diagnosis had already severely impaired hemodynamic profile (mPAP > 35 mmHg, cardiac index of less than 3 L/min/m^2^). In the same study, the 3-year survival in NYHA-FC II patients was 80%, higher than previously reported (>66%) by the UK PH research group [74].

In a recent subgroup analysis of the largest known to date US cohort of RHC-confirmed PH patients [77] (REVEAL study), SSc-PH patients did not differ in hemodynamics at the initial diagnostic RHC compared to other CTDs, with an exception in the right atrial pressure (RAP) (SSc-PH group RAP was 9.1 ± 5.9 versus 8.1 ± 5.0 mmHg, *P* = 0.05). In addition, a higher percentage of patients with SSc-PH were in NYHA-FC IV at the time of enrollment, compared with patients suffering from other CTDs (*P* = 0.04), but the 6-minute walking distance test (6MWD) was not significantly different. In relation to pulmonary arterial pressure estimates by TTE at the time of enrollment, SSc-PH patients were significantly better than in other CTDs, with a lower percentage of RV enlargement and LV systolic dysfunction. The 1-year survival for SSc-PH patients was 87%, comparing to 93% of IPAH. In a 3-year survival followup, rates dropped to 47% for SSc-PH. Having in mind that these patients were treated under the current guidelines, their high 3-year mortality raises questions about the effectiveness of their current management. It should be noted that systemic sclerosis associated PH may be multifactorial: true PAH, left heart disease associated PH, and ILD-associated PH might sometimes overlap in the same patient.

### 4.3. Systemic Lupus Erythematosus and Pulmonary Hypertension

Systemic lupus erythematosus (SLE) is a complicated autoimmune disease of unclear pathogenesis, affecting multiple organs [78]. The pulmonary involvement, which results in SLE-PH, appears commonly in adult patients.

The theory of vasculitis, *in situ *thrombosis, and SMCs proliferation also applies in SLE-PH pathophysiology, with the exact causal relationship being still under investigation [79–82]. Several factors are incriminated for the induction of SLE-PH, such as hypoxic vasoconstriction, pulmonary venous hypertension resulting from left heart disease, antiphospholipid antibody-induced chronic or acute thrombosis, and pulmonary venoocclusive disease (PVOD) [83–88]. There are several pathological similarities in SLE-PH and IPAH, including SMCs hypertrophy, hyperactivation of transcription factors like hypoxia inducible factor-1 alpha and nuclear factor of activated T-lymphocytes, decreased expression of certain voltage-gated potassium channels, and *de novo* expression of antiapoptotic proteins [89]. Interestingly, immunoglobulin and complement deposition has been found in the pulmonary arterial wall of SLE patients [90]. In addition, mitral and aortic valve damage (also known as Libman-Sacks endocarditis) might occur in SLE patients, cause regurgitation, and subsequently provoke pulmonary venous hypertension. The exact incidence of this complication has not been effectively determined.

The prevalence of SLE-PH is largely unknown, with unconfirmed data reporting it from 0.5% to 14% in adults, in whom it is commonly associated with Raynaud's phenomenon [91, 92], and in childhood-onset SLE approximately 4%–8% using TTE assessment [93]. In a study by Prabu et al. [94] in SLE patients assessed by TTE, the prevalence of PH was lower than it usually appears (4.2%), and only 3 of the 12 study patients were found to have high sPAP (>40 mmHg). Although the study sample was very small, these results are worth noting because of the study population, which, in contrast to other studies, had a community nontertiary background and therefore might be considered as vicarious of the general SLE population.

### 4.4. Rheumatoid Arthritis and Pulmonary Hypertension

Rheumatoid arthritis (RA) is a chronic, systemic autoimmune inflammatory disease, affecting 1% of the general population and over 5% in ages >65 years. Besides its articular manifestations, RA can cause severe disability, with multiple extra-articular insults in over 40% of all RA patients, including the lung, with ILD being the most common manifestation in this organ [95–98].

Incidence of RA-PH is rather unknown, and the largest up-to-date study by Dawson et al. (*n* = 146) reported that 21% of the cohort had mild-to-moderate PH as assessed by TTE, while 19% of all patients enrolled had sPAP values within the 30–35 mmHg range. Major limitation in this study was the low cut-off point selection for sPAP (30 mmHg), which might have resulted in overestimating a considerable number of RA patients that were in the “grey zone” and might have led to precarious results [95].

### 4.5. Sjögren Syndrome and Pulmonary Hypertension

This is a chronic inflammatory disorder characterized by diminished lacrimal and salivary gland function and associated with lymphocytic infiltration of exocrine glands, especially the lacrimal and salivary glands. Sjögren syndrome (SS) also affects extraglandular systems such as skin, lung, heart, kidney, neural, and hematopoietic system. It can be seen in a sole form as a primary disorder (primary SS) or in the onset of an associated rheumatic disease (RA, SLE, SSc) with a peak among women >50 years of age [99]. The major complaints are skin dryness, xerostomia (mouth dryness), and keratoconjunctivitis sicca (dry eyes). In primary SS, there is a subclinical lung inflammatory process in more than 50% of patients, but interestingly, only 1 in 5 develops clinically significant pulmonary disease. Lung insult can be multiple, with a variety of manifestations such as xerotrachea and bronchial sicca (dryness in the tracheobronchial tree), obstructive small airway disease, ILD, lymphocytic interstitial pneumonitis (LIP), pleural effusions, lung cysts, thromboembolic disease, and PH [100].

Pathogenesis of PH in SS remains a clinical enigma. Drawing on data from a small number of reported cases (45 overall, since 1982; PubMed search June 27, 2012), patients with SS-associated PH (SSPH) have Reynaud's, cutaneous vasculitis, and ILD more frequently, compared to SS patients without PH. In addition, they seem to have quite frequent detectable antinuclear, anti-Ro/SSA, and anti-RNP autoantibodies, as well as positive rheumatoid factor and hypergammaglobulinemia. In summarized data available from 32 out of the 45 overall reported cases, patients' functional status was found to be markedly impaired (NYHA-FC III and IV in most cases), and so were their hemodynamics (mPAP = 44 ± 11 mmHg, CI 2.91 ± 0.72 L/min/m^2^) [101]. These findings, although punctuating the data insufficiency in this field, might suggest that systemic vasculopathy, activation of B-cells, and autoimmunity could be factors in the SSPH disease process.

## 5. Sarcoidosis and Pulmonary Hypertension

Sarcoidosis is a chronic, systemic granulomatous inflammatory disease that can affect any organ [102]. Although there is massive progress during the past decade, the pure pathogenesis of sarcoidosis is still undistinguished.

Sarcoidosis-associated PH (SA-PH) is one of the trickiest to define and lies in group 5.2 (PH with unclear and/or multifactorial mechanisms/systemic disorders) in current Dana Point PH classification, mainly because of its heterogeneity and lack of data, although this specific categorization has been criticized [103]. The main criticism is that sarcoidosis should be included in group 3 (PH owing to lung disease and/or hypoxia), along with pulmonary Langerhans cells histiocytosis (PLCH) and lymphangioleiomyomatosis (LAM), which are currently also classified in group 5.2, based on the fact that PH in such cases mainly occurs due to massive lung involvement and profound hypoxia [104, 105].

Several pathogenic mechanisms are implicated in SA-PH development, with major causal factor the destruction of distal capillaries due to fibrosis that leads to chronic hypoxia, increased PVR, and pulmonary arterial pressure [106–108]. Vascular involvement is quite established in pulmonary sarcoidosis, with a reported occurrence of 69% to 100% in pathological-histological case studies [109, 110]. However, it is of interest that SA-PH has already been stated as an early complication in the disease course. In addition, there is no reported correlation with the severity of SA-PH and the grade of lung fibrosis. These findings could suggest that other mechanisms might contribute to PH development in such patients, such as “outside” compression by mediastinal and hilar lymphadenopathy on main pulmonary arteries or their large branches [111], vascular granulomatous involvement [112] with the possibility of secondary PVOD development, and pulmonary vasoconstriction induced by vasoactive agents [113]. In certain cases, portal hypertension due to liver sarcoidosis can also cause PH mediated by increased circulating endothelin-1 (ET-1) levels [114].

The exact prevalence of SA-PH is not known, partly because of the population selection in several studies and their different diagnostic protocols. Recently, two separate single-center studies, concerning SA-PH development in consecutive patients suffering from sarcoidosis, reported an incidence of 5% to 15% [115, 116]. In other cohorts enrolling symptomatic-only sarcoidosis patients, the prevalence of SA-PH was higher than 50% [117, 118]. The highest prevalence documented by RHC has been reported in patients listed for lung transplantation (74%), with a concurrent increase in mortality rate, compared to listed patients without PH [111].

## 6. Idiopathic Pulmonary Fibrosis (IPF) and Pulmonary Hypertension

IPF is an idiopathic, fibrosing, interstitial, chronic lung disease with a characteristic appearance in histological findings currently known as usual interstitial pneumonia (UIP). It involves abnormal collagen deposition in the pulmonary interstitium (alveoli walls) with an associated inflammation. IPF has been linked to cigarette smoking and gastroesophageal reflux disease, but these factors are not present in all IPF patients. Genetic associations with the disease include pulmonary surfactant-associated proteins (SFTPA-1 and SFTPA-2), telomerase reverse transcriptase (TERT), and telomerase RNA component (TERC) [119, 120]. It is of interest that statistically significant association in survival has been reported between IPF patients with and without PH at the time of initial IPF diagnosis [121]; PH in IPF can develop either as consequence of the fibrotic process or disproportionate to the degree of fibrotic lung damage [122]. Although chronic hypoxia and its subsequent pulmonary arterial vasoconstriction are thought to have a major role in secondary IPF-PH, studies that showed the existence of PH in such patients even with arterial pO_2_ levels within normal range (normoxic) led the investigators to partly relinquish this concept and redirect to other possible underlying mechanisms [123–125]. However, in one study of 70 IPF patients, there was a significant, but rather loose, correlation between mPAP and both PaO_2_ and DLCO (*R* = −0.47, *P* < 0.001 and *R* = −0.46, *P* < 0.001, resp.) [126].

In “out-of-proportion” to the degree of fibrotic lung damage IPF-PH, there is a much more complex mechanism involved. Taking into account the extensive alveolar damage, the growth of connective tissue, and the ongoing inflammatory process in IPF, vascular remodeling of pulmonary arteries might be more important in the development of “out-of-proportion” IPF-PH than hypoxic vasoconstriction. In favor of this perspective, there is an inconsistency in PH severity and pathological findings; reduction in vessel density and vascular ablation in IPF patients have been reported, especially in “honeycombing” areas, along with simultaneous development of new vessels (neoangiogenesis) [127–132].

Furthermore, there is data regarding the role of endothelial cell dysfunction in “out-of-proportion” IPF-PH, also justifying the bad correlation between the severity of lung fibrosis and PH development. A microarray gene study involving a subgroup of IPF-PH patients revealed an unexpected underexpression of genes such as the vascular endothelial growth factor (EGF), the platelet endothelial cell adhesion molecule (PECAM), as well as factors known to regulate vascular tone, such as ACE and ET-1 (*P* < 0.05) [133]. In contrast, an overexpression of the phospholipase A2 gene was noticed, which could be potentially causative in pulmonary vascular remodeling [133].

Interestingly, several mediators that are established in IPAH have been recently incriminated for “out-of-proportion” IPF-PH. Such mediators are tumor necrosis factor alpha (TNF-*α*), platelet-derived growth factor (PDGF), and fibroblast growth factor [134]. Additionally, studies on the role of eicosanoids both in IPF and PH suggest a potential role of supplementation of PGE2 or prostacyclin in IPF patients, particularly those with PH [134]. The basic postulant in “out-of-proportion” IPF-PH pathogenesis may be ET-1, as suggested by recent data revealing a profibrotic ability of ET-1 in patients with IPF but no clinical evidence of PH [135]. Levels of ET-1 have also been found elevated in airway epithelium, type-2 pneumocytes, and pulmonary vascular endothelial cells [136–138]. In currently published experimental data, PDGF is under investigation as a potential therapeutic target in IPF and it is of interest that it has been found upregulated in PH. Furthermore, tissue growth factor beta (TGF-*β*), which is a possible pathogenetic cytokine of interstitial fibrosis, showed impaired signaling in patients with IPAH and could be another underlying mediator in pulmonary vascular remodeling in “out-of-proportion” IPF-PH [139].

Epidemiologically, both types of IPF-PH combined (secondary and “out-of-proportion”) affect a large number of patients with IPF, especially those who are listed for lung transplantation. The prevalence of PH in all IPF patients shows a wide range, being reported from 14.2% to 84% [124, 140].

This large variation in reported prevalence values might be related, at least in part, to the method of PAP measurement (estimated sPAP in TTE or exact mPAP in RHC), the difference in selected pressure cut-off value, and to the timing of measurement. In recently published data, it was suggested that a key point in such patient cohorts seems to be “how fast” PH progresses in time, and not “how severe” PH is on a single time point of sPAP estimation by TTE or mPAP measurement by RHC [140–142]. The presence of PH confirmed by RHC in IPF lung transplanted patients preoperatively has a negative effect on survival and notably increases the risk for developing primary graft dysfunction (PGD) in the posttransplantation period; for every increase of 10 mmHg in mPAP, the odds of PGD increase by 1.64 (CI 95%, 1.18–2.26; *P* = 0.003) [141]. One study underlined a PH prevalence of 33% in the initial RHC measurements that jumped to 85% in the pretransplantation assessment. In another study, baseline prevalence was 41% and jumped to 90% in the follow-up RHC measurement [140, 142]. The question of whether the lung fibrotic process and the vascular alterations that lead to PH share common pathophysiologic pathways remains open.

It should be noted that possible treatment options in IPF-PH by means of PAH-specific agents have been tested; disappointingly, 3 large RCTs, where the dual endothelin receptor antagonist (ERA) bosentan and the phosphodiesterase-5 inhibitor sildenafil were used, gave negative results [26, 31, 32].

## 7. Lymphangioleiomyomatosis (LAM) and Pulmonary Hypertension

It is a multisystemic disease, affecting mostly young women and characterized by abnormal SMC deposition along lymphatics of the thorax and abdomen. As a result, there is a formation of lung cysts and abdominal tumors, predominantly renal angiomyolipomas [143–148]. As far as it concerns the lung, LAM decreases FEV_1_ and DLCO, with the latter previously demonstrated as an independent predictor of mortality in patients listed for lung transplantation, and aggravates peak oxygen uptake (VO_2_ max) [149, 150].

The pathogenesis of PH in LAM (LAM-PH) is quite complex and not completely clarified. As in other lung diseases, chronic hypoxia resulting from the damaged lung parenchyma (i.e., cyst formation) can cause pulmonary hypoxic vasoconstriction and increase PVR, trigger the vascular remodeling process, and establish PH. However, in LAM patients, there is a low reported observation of RV failure and high PAP at rest, suggestive of different pathway(s) [151]. Taveira-DaSilva et al. evaluated a cohort of LAM patients for PH, estimating resting and exercise PAP with TTE, under cardiopulmonary exercise testing (CPET). Overall, resting TTE-estimated sPAP was found to be 26 ± 0.7 mmHg, while exercise TTE-estimated sPAP was 40.5 ± 1.1 mmHg. Resting LAM-PH was present in less than 10% of the cohort (8 out of 95, sPAP = 43 ± 3 mmHg) [151]. In recently published data extracted from patients with severe disease, listed for lung transplantation and evaluated by RHC, morphological and clinical signs of PH were present in all subjects [152]. Similarly, another recent retrospective multicenter study reported data from RHC evaluations in LAM-PH patients. Severe PH (defined by the investigators as mPAP > 35 mmHg) was present in only 20% of patients. Interestingly, 6 patients received oral PAH specific therapy and improved hemodynamically (mPAP decreased from 33 ± 9 to 24 ± 10 mmHg and PVR from 481 ± 188 to 280 ± 79 dyn/s/cm^−5^). In this cohort of 20 female patients, the overall 2-year survival was 94% [153]. There is very few available data regarding LAM-PH, and the field needs more large-scale studies to extract more enlightening data regarding pathophysiology and prevalence of disease.

## 8. Chronic Obstructive Pulmonary Disease and Pulmonary Hypertension

The pathogenesis of “out-of-proportion” PH in COPD (COPD-PH) is quite complex and being continuously elucidated by ongoing research. Pulmonary vascular endothelial dysfunction, as well as the inflammatory effect, is roughly the outline of the disease mechanisms. A major inflammatory factor in COPD is thought to be tobacco smoke inhalation, with established vascular and parenchymal changes in human and experimental animal lungs, and could act additively in COPD-PH as a direct hit to pulmonary vasculature [154, 155]. There is a documented decrease of endothelial NO synthase (eNOS) expression and impaired vasodilation response in asymptomatic smokers, as well as in advanced COPD disease, delineating a potential role of eNOS in the disease [156–160]. Additionally, certain eNOS and ACE polymorphisms have been found to be associated with COPD-PH [161]. Interleukin-6 (IL-6) and the presence of its polymorphism were associated with higher PAP in COPD patients, adumbrating an involvement in COPD-PH pathogenesis [162, 163].

At first, as in other parenchymal lung disease-associated PH subtypes, acute hypoxia-induced vasoconstriction was thought to be the initializing factor in vascular remodeling. In fact, chronic hypoxia induces the neomuscularization of pulmonary arterioles, resulting in intimal thickening by SMC assemblage and extracellular deposition of plenteous collagen and elastin, a phenomenon widely referred as “intimal fibroelastosis.” Of great interest is that these changes have also been described in normoxemic (pO_2_ within normal range) COPD patients without pulmonary hypertension and also in asymptomatic smokers [159]. In addition, in an experimental animal study, pCO_2_ as well as pH was found to have an amplifying effect on acute hypoxia-induced vasoconstriction [164]. 

Recent data proposes an important role for serotonin (5-HT) and its transporter (5-HTT) in intimal fibroelastosis. The 5-HTT LL genotype, which is linked with greater 5-HTT expression, was found to be associated with considerably high PAP in COPD, compared to other polymorphisms [165]. A pathological examination of postpneumonectomy lungs demonstrated mass attraction of mostly CD8+ lymphocytes infiltrating the vascular adventitia [166].

An adaptive response to hypoxemia is polycythemia (increased total erythrocyte number), which is also incriminated for alterations in pulmonary vasculature. It has been shown experimentally that a sole hematocrit increment in dogs can notably increase PVR by 112% (*P* < 0.01). Moreover, there was a combined augmentation effect of polycythemia and hypoxia, increasing PVR by 308% (*P* < 0.005) [167]. Recently, it was demonstrated that the presence of excessive erythrocytosis in mice increased the sPAP *in vivo* [168]. Additive data shows that there is definitely a role of polycythemia in the COPD-PH mechanism, but in humans is yet to be investigated. 

The true incidence of clinically significant resting “out-of-proportion” PH is difficult to be estimated in COPD patients, as most data comes from reports that include COPD patients with advanced disease, resulting in a notably wide reported range varying from 5% to 70% [169–171]. This is cofounded by several limitations. Firstly, there are no large-scale studies assessing the true prevalence of COPD-PH by means of RHC. Commonly, the test selected for PH documentation in such patients is TTE. As already emphasized elsewhere in this paper, TTE can only estimate sPAP and mPAP values, and only the invasive RHC can establish the presence of elevated PAP. This must be kept in mind by the clinician when evaluating the reported incidence for COPD-PH, because in many settings PH diagnosis relies only on TTE. There is additive data for this statement, showing TTE inaccuracy in PAP and cardiac output (CO) estimation, when compared to RHC, in several PH subtypes [172]. Secondly, most available studies are of retrospective nature and include mostly patients with severe disease (FEV_1_ < 30% predicted). As an example, studies on severe COPD patients report an incidence of 91%, with the majority suffering from mild-to-moderate PH (mPAP = 20–35 mmHg) and 1% to 5% suffering from severe disease (mPAP > 35–40 mmHg) [8, 169, 173]. However, in some COPD patients, the hemodynamic impairment might be more severe than expected from the related progress of parenchymal disease. This group of patients is characterized in anecdotal basis as “PH out-of-proportion to degree of respiratory compromise.” This is of significant interest, because such patients have been viewed as potential beneficiaries of PAH-specific therapeutic agents, although, as of now, there is neither consensus on the best candidates for studying such management, nor RCTs running. 

It seems that there is a strong negative impact on survival from the occurrence of PH in COPD patients, even though the hemodynamic impairment is rather mild in terms of pressure values *per se*. The 5-year survival regarding severely affected COPD patients with PH (mPAP ≥ 25 mmHg) has been reported as low as 36%, compared to 62% in COPD patients without PH [174]. Although several studies demonstrate high mortality rates in COPD patients with pulmonary hypertension, it is still under discussion if the occurrence of pulmonary hypertension is an independent cause of death or just a sign of disease worsening.

## 9. Treatment Suggestions for Pulmonary Hypertension in Lung Disease

It should be emphasized that specific treatment for PAH has been approved by regulatory authorities for group 1 (PAH) only [1]. Drug-related information provided herein (text and Table 1) is based on case reports and small case series, provided to roughly inform the reader about current anecdotal use of PAH-specific agents in selected cases. This results in minimum strength of evidence, and the need for large-scale randomized controlled trials is profound.

Patients with underlying parenchymal lung diseases who develop PH are always an intriguing subset regarding their management and treatment, as the occurrence of PH is associated with mortality; whether this association has a causal relation with mortality or simply represents a marker of disease severity is not clear. 

There is no clear consensus on how or when to treat severe PH in parenchymal lung diseases. PAH-specific treatment in this setting does not ensure improvement of pulmonary vascular hemodynamics or exercise capacity while on the other hand might worsen ventilation/perfusion (V/Q) mismatch and subsequently lead to shunting and further hypoxia [27, 33, 37].

As of today, the European guidelines regarding “out-of-proportion” PH (PH owing to lung disease and/or hypoxia) recommend performance of TTE for screening (Class of recommendation-Level of evidence, I-C) and RHC for a definite diagnosis of PH due to lung disease (I-C). Again, the use of PAH-specific therapeutic agents is not recommended in this group (III-C). Additionally, optimal treatment of the underlying lung disease and the use of supplemental O_2_ are the recommended therapeutic measures in such patients. In PAH associated with CTDs, the recommendation is for the same treatment algorithm as in IPAH (I-A); *terra incognita* remains the group of CTD patients with significant ILD, since such patients have usually been excluded from performed related RCTs. The performance of TTE is strongly recommended in all symptomatic patients with scleroderma for PAH screening (I-C) and RHC is recommended in all patients with the clinical question of starting a PAH-specific treatment (I-C). In nonsymptomatic patients with scleroderma, a screening study (TTE) may be considered (IIb-C) [1].

In conclusion, we emphasize again that the use of PAH-specific therapeutic agents is not approved for patients belonging to groups 3 and 5 by the Dana Point classification [1], which is the case of all the diseases analyzed in this review with the exception of CTDs. Clinical studies and RCTs should be performed in such nongroup 1 patients, in an effort to clearly designate subcategories of subjects that might benefit from specific treatments. 

## Figures and Tables

**Table 1:** Representative randomized control trials and studies on non-PAH pulmonary hypertension related to parenchymal lung diseases.

Treatment	Lung disease	Study/reference	Comments
Sildenafil	Lung fibrosis including an IPF subgroup	Ghofrani et al., 2002, [22]	Improvement in hemodynamics and gas exchange
Sildenafil	IPF	Collard et al., 2007, [23]	Improvement in 6MWD in 57% of patients
Sildenafil	IPF	Jackson et al., 2010, [24]	No improvement in 6MWD
Sildenafil	IPF	Madden et al., 2006, [25]	Only 3 patients treated for 3 months and showed improvement in 6MWD and TTE parameters
Sildenafil	IPF	The IPF Clinical Research Network, 2010, [26]	There was no difference in 6MWD between the two groups, as a primary outcome measure
Sildenafil	COPD	Rietema et al., 2008, [27]	No improvement in stroke volume or exercise capacity
Sildenafil	Sarcoidosis	Barnett et al., 2009, [28]	In 9 patients treated with sildenafil out of 22 total, there was slight improvement in hemodynamics, 6MWD, and NYHA-FC
Sildenafil	Sarcoidosis	Milman et al., 2008, [29]	In 12 patients treated, who were listed for transplantation, there was a significant decrease in mPAP. No improvement found in 6MWD
Sildenafil	COPD	Blanco et al., 2010, [30]	In a RCT of 20 patients with COPD-associated PH, sildenafil improved acute pulmonary hemodynamics at rest and during exercise and deteriorated oxygenation
Bosentan	IPF	BUILD-1 study, King et al., 2008, [31]	Bosentan treatment in patients with IPF did not show superiority over placebo on 6MWD
Bosentan	IPF	BUILD-3 study, King et al., 2011, [32]	No treatment effects were observed on health-related quality of life or dyspnea. The primary objective was not met
Bosentan	COPD	Stolz et al., 2008, [33]	30 patients with COPD were randomly assigned in a 2 : 1 ratio to receive either bosentan or placebo for 12 weeks. Bosentan did not improve 6MWD and deteriorated hypoxemia and functional class
Bosentan	COPD	Valerio et al., 2009, [34]	In a quite small sample size (*n* = 16), there was benefit in PAP, PVR, and 6MWD. No improvement in GOLD IV patients
Riociguat	COPD	Ghofrani et al., 2011, [35]	In a quite small sample size (*n* = 22), there was a trend of improvement in hemodynamics (abstract)
IV epoprostenol	Sarcoidosis	Fisher et al., 2006, [36]	In 5 patients treated with parenteral epoprostenol, there was improvement of NYHA-FC by one or two stages within 29 months
IV prostacyclin	COPD	Archer et al., 1996, [37]	Treatment of 7 mechanically ventilated patients for COPD exacerbation caused worsening of hypoxemia

6MWD: 6-minute walking distance test; COPD: chronic obstructive pulmonary disease; INH: inhaled; IPF: idiopathic pulmonary fibrosis; IV: intravenous; mPAP: mean pulmonary artery pressure; NYHA-FC: New York Heart Association functional class; PAP: pulmonary artery pressure; PVR: pulmonary vascular resistance; TTE: transthoracic tissue Doppler echocardiography.

## References

[1] Galie N, Hoeper MM, Humbert M (2009). Guidelines for the diagnosis and treatment of pulmonary hypertension: the Task Force for the Diagnosis and Treatment of Pulmonary Hypertension of the European Society of Cardiology (ESC) and the European Respiratory Society (ERS), endorsed by the International Society of Heart and Lung Transplantation (ISHLT). *European Heart Journal*.

[2] Humbert M, Sitbon O, Chaouat A (2006). Pulmonary arterial hypertension in France: results from a national registry. *American Journal of Respiratory and Critical Care Medicine*.

[3] Peacock AJ, Murphy NF, McMurrey JJV, Caballero L, Stewart S (2007). An epidemiological study of pulmonary arterial hypertension. *European Respiratory Journal*.

[4] D’Alonzo GE, Barst RJ, Ayres SM (1991). Survival in patients with primary pulmonary hypertension: results from a national prospective registry. *Annals of Internal Medicine*.

[5] McGoon M, Gutterman D, Steen V (2004). Screening, early detection, and diagnosis of pulmonary arterial hypertension: ACCP evidence-based clinical practice guidelines. *Chest*.

[6] Houtchens J, Martin D, Klinger JR (2011). Diagnosis and management of pulmonary arterial hypertension. *Pulmonary Medicine*.

[7] Ling Y, Johnson MK, Kiely DG Changing demographics, epidemiology and survival of incident pulmonary arterial hypertension.

[8] Thabut G, Dauriat G, Stern JB (2005). Pulmonary hemodynamics in advanced COPD candidates for lung volume reduction surgery or lung transplantation. *Chest*.

[9] Chaouat A, Bugnet AS, Kadaoui N (2005). Severe pulmonary hypertension and chronic obstructive pulmonary disease. *American Journal of Respiratory and Critical Care Medicine*.

[10] Hoeper M, Andreas S, Bastian A (2011). Pulmonary hypertension due to chronic lung disease: updated recommendations of the Cologne consensus conference 2011. *International Journal of Cardiology*.

[11] Gabbay E, Yeow W, Playford D (2007). Pulmonary arterial hypertension (PAH) is an uncommon cause of pulmonary hypertension (PH) in an unselected population: the Armadale echocardiography study. *American Journal of Respiratory and Critical Care Medicine*.

[12] Dumas JP, Bardou M, Goirand F, Dumas M (1999). Hypoxic pulmonary vasoconstriction. *General Pharmacology*.

[13] Orfanos SE, Mavrommati I, Korovesi I, Roussos C (2004). Pulmonary endothelium in acute lung injury: from basic science to the critically ill. *Intensive Care Medicine*.

[14] Tuder RM, Davis LA, Graham BB (2012). Targeting energetic metabolism: a new frontier in the pathogenesis and treatment of pulmonary hypertension. *American Journal of Respiratory and Critical Care Medicine*.

[15] Dorfmüller P, Humbert M, Perros F (2007). Fibrous remodeling of the pulmonary venous system in pulmonary arterial hypertension associated with connective tissue diseases. *Human Pathology*.

[16] Fagan KA, Fouty BW, Tyler RC (1998). The pulmonarycirculation of mice with either homozygous or heterozygous disruption of endothelial nitric oxide synthase is hyperresponsive to chronic mild hypoxia. *Journal of Clinical Investigation*.

[17] Tuder RM, Groves B, Badesch DB, Voelkel NF (1994). Exuberant endothelial cell growth and elements of inflammation are present in plexiform lesions of pulmonary hypertension. *American Journal of Pathology*.

[18] Peacock AJ (1999). Primary pulmonary hypertension. *Thorax*.

[19] Wideman RF, Hamal KR (2011). Idiopathic pulmonary arterial hypertension: an avian model for plexogenic arteriopathy and serotonergic vasoconstriction. *Journal of Pharmacological and Toxicological Methods*.

[20] Yousem SA (1990). The pulmonary pathologic manifestations of the CREST syndrome. *Human Pathology*.

[21] Lee SD, Shroyer KR, Markham NE, Cool CD, Voelkel NF, Tuder RM (1998). Monoclonal endothelial cell proliferation is present in primary but not secondary pulmonary hypertension. *Journal of Clinical Investigation*.

[22] Ghofrani HA, Wiedemann R, Rose F (2002). Sildenafil for treatment of lung fibrosis and pulmonary hypertension: a randomised controlled trial. *The Lancet*.

[23] Collard HR, Anstrom KJ, Schwarz MI, Zisman DA (2007). Sildenafil improves walk distance in idiopathic pulmonary fibrosis. *Chest*.

[24] Jackson RM, Glassberg MK, Ramos CF, Bejarano PA, Butrous G, Orlando Gómez-Marín O (2010). Sildenafil therapy and exercise tolerance in idiopathic pulmonary fibrosis. *Lung*.

[25] Madden BP, Allenby M, Loke TK, Sheth A (2006). A potential role for sildenafil in the management of pulmonary hypertension in patients with parenchymal lung disease. *Vascular Pharmacology*.

[26] Zisman DA, Schwarz M, Anstrom KJ, Collard HR, Flaherty KR, Hunninghake GW (2010). A controlled trial of sildenafil in advanced idiopathic pulmonary fibrosis. *The New England Journal of Medicine*.

[27] Rietema H, Holverda S, Bogaard HJ (2008). Sildenafil treatment in COPD does not affect stroke volume or exercise capacity. *European Respiratory Journal*.

[28] Barnett CF, Bonura EJ, Nathan SD (2009). Treatment of sarcoidosis-associated pulmonary hypertension: a two-center experience. *Chest*.

[29] Milman N, Burton CM, Iversen M, Videbæk R, Jensen CV, Carlsen J (2008). Pulmonary hypertension in end-stage pulmonary sarcoidosis: therapeutic effect of sildenafil?. *Journal of Heart and Lung Transplantation*.

[30] Blanco I, Gimeno E, Munoz PA (2010). Hemodynamic and gas exchange effects of sildenafil in patients with chronic obstructive pulmonary disease and pulmonary hypertension. *American Journal of Respiratory and Critical Care Medicine*.

[31] King TE, Behr J, Brown KK (2008). BUILD-1: a randomized placebo-controlled trial of bosentan in idiopathic pulmonary fibrosis. *American Journal of Respiratory and Critical Care Medicine*.

[32] King TE, Brown KK, Raghu G (2011). BUILD-3: a randomized placebo-controlled trial of bosentan in idiopathic pulmonary fibrosis. *American Journal of Respiratory and Critical Care Medicine*.

[33] Stolz D, Rasch H, Linka A (2008). A randomised, controlled trial of bosentan in severe COPD. *European Respiratory Journal*.

[34] Valerio G, Bracciale P, Grazia D’Agostino A (2009). Effect of bosentan upon pulmonary hypertension in chronic obstructive pulmonary disease. *Therapeutic Advances in Respiratory Disease*.

[35] Ghofrani HA, Staehler G, Gruenig E (2011). The effect of the soluble guanylate cyclase stimulator Riociguat on hemodynamics in patients with pulmonary hypertension due to chronic obstructive pulmonary disease. *American Journal of Respiratory and Critical Care Medicine*.

[36] Fisher KA, Serlin DM, Wilson KC, Walter RE, Berman JS, Farber HW (2006). Sarcoidosis-associated pulmonary hypertension: outcome with long-term epoprostenol treatment. *Chest*.

[37] Archer SL, Mike D, Crow J, Long W, Weir EK (1996). A placebo-controlled trial of prostacyclin in acute respiratory failure in COPD. *Chest*.

[38] Roberts JD, Forfia PR (2011). Diagnosis and assessment of pulmonary vascular disease by Doppler echocardiography. *Pulmonary Circulation*.

[39] Lang IM, Plank C, Sadushi-Kolici R, Jakowitsch J, Klepetko W, Maurer G (2010). Imaging in pulmonary hypertension. *Cardiovascular Imaging*.

[40] Hammerstingl C, Schueler R, Bors L (2012). Diagnostic value of echocardiography in the diagnosis of pulmonary hypertension. *PLoS ONE*.

[41] Farber HW, Foreman AJ, Miller DP, Mcgoon MD (2011). REVEAL registry: correlation of right heart catheterization and echocardiography in patients with pulmonary arterial hypertension. *Congestive Heart Failure*.

[42] Galiè N, Hoeper MM, Humbert M (2009). Guidelines for the diagnosis and treatment of pulmonary hypertension. *European Respiratory Journal*.

[43] Galiè N, Manes A, Farahani KV (2005). Pulmonary arterial hypertension associated to connective tissue diseases. *Lupus*.

[44] Hachulla E, de Groote P, Gressin V (2009). Itinér AIR-Sclérodermie Study Group. The three-year incidence of pulmonary arterial hypertension associated with systemic sclerosis in a multicenter nationwide longitudinal study in France. *Arthritis and Rheumatism*.

[45] Steen VD, Medsger TA (2007). Changes in causes of death in systemic sclerosis, 1972–2002. *Annals of the Rheumatic Diseases*.

[46] Mukerjee D, St George D, Coleiro B (2003). Prevalence and outcome in systemic sclerosis associated pulmonary arterial hypertension: application of a registry approach. *Annals of the Rheumatic Diseases*.

[47] Wigley FM, Lima JAC, Mayes M, McLain D, Chapin JL, Ward-Able C (2005). The prevalence of undiagnosed pulmonary arterial hypertension in subjects with connective tissue disease at the secondary health care level of community-based rheumatologists (the UNCOVER study). *Arthritis and Rheumatism*.

[48] Owens GR, Fino GJ, Herbert DL (1983). Pulmonary function in progressive systemic sclerosis. Comparison of CREST syndrome variant with diffuse scleroderma. *Chest*.

[49] Simonneau G, Galie N, Rubin LJ (2004). Clinical classification of pulmonary hypertension. *Journal of the American College of Cardiology*.

[50] Humbert M, Sitbon O, Simonneau G (2004). Treatment of pulmonary arterial hypertension. *The New England Journal of Medicine*.

[51] Orfanos SE, Langleben D (2010). Pulmonary arterial hypertension in systemic sclerosis: a distinctive endotheliopathy?. *European Respiratory Journal*.

[52] Overbeek MJ, Vonk MC, Boonstra A (2008). Pulmonary arterial hypertension in limited cutaneous systemic sclerosis: a distinctive vasculopathy. *European Respiratory Journal*.

[53] Langleben D, Orfanos SE, Giovinazzo M (2008). Pulmonary capillary endothelial metabolic dysfunction: severity in pulmonary arterial hypertension related to connective tissue disease versus idiopathic pulmonary arterial hypertension. *Arthritis and Rheumatism*.

[54] Orfanos SE, Psevdi E, Stratigis N (2001). Pulmonary capillary endothelial dysfunction in early systemic sclerosis. *Arthritis and Rheumatism*.

[55] Young ID, Ford SE, Ford PM (1989). The association of pulmonary hypertension with rheumatoid arthritis. *Journal of Rheumatology*.

[56] Balagopal VP, da Costa P, Greenstone MA (1995). Fatal pulmonary hypertension and rheumatoid vasculitis. *European Respiratory Journal*.

[57] Dalakas MC, Hohlfeld R (2003). Polymyositis and dermatomyositis. *The Lancet*.

[58] Bohan A, Peter JB (1975). Polymyositis and dermatomyositis-I. *The New England Journal of Medicine*.

[59] Bohan A, Peter JB (1975). Polymyositis and dermatomyositis (Second of two parts). *The New England Journal of Medicine*.

[60] Torres C, Belmonte R, Carmona L (2006). Survival, mortality and causes of death in inflammatory myopathies. *Autoimmunity*.

[61] Chinoy H, Salway F, Fertig N (2006). In adult onset myositis, the presence of interstitial lung disease and myositis specific/associated antibodies are governed by HLA class II haplotype, rather than by myositis subtype. *Arthritis Research and Therapy*.

[62] O’Hanlon TP, Carrick DM, Arnett FC (2005). Immunogenetic risk and protective factors for the idiopathic inflammatory myopathies: distinct HLA-A, -B, -Cw, -DRB1 and -DQA1 allelic profiles and motifs define clinicopathologic groups in Caucasians. *Medicine*.

[63] O’Hanlon TP, Carrick DM, Targoff IN (2006). Immunogenetic risk and protective factors for the idiopathic inflammatory myopathies: distinct HLA-A, -B, -Cw, -DRB1, and -DQA1 allelic profiles distinguish European American patients with different myositis autoantibodies. *Medicine*.

[64] Targoff IN (2002). Laboratory testing in the diagnosis and management of idiopathic inflammatory myopathies. *Rheumatic Disease Clinics of North America*.

[65] Minai OA (2009). Pulmonary hypertension in polymyositis-dermatomyositis: clinical and hemodynamic characteristics and response to vasoactive therapy. *Lupus*.

[66] Yaqub S, Moder KG, Lacy MQ (2004). Severe, reversible pulmonary hypertension in a patient with monoclonal gammopathy and features of dermatomyositis. *Mayo Clinic Proceedings*.

[67] Denbow CE, Lie JT, Tancredi RG, Bunch TW (1979). Cardiac involvement in polymyositis. A clinicopathologic study of 20 autopsied patients. *Arthritis and Rheumatism*.

[68] Fathi M, Lundberg IE (2005). Interstitial lung disease in polymyositis and dermatomyositis. *Current Opinion in Rheumatology*.

[69] Richards TJ, Eggebeen A, Gibson K (2009). Characterization and peripheral blood biomarker assessment of anti-Jo-1 antibody-positive interstitial lung disease. *Arthritis and Rheumatism*.

[70] Gabrielli A, Avvedimento EV, Krieg T (2009). Mechanisms of disease: scleroderma. *The New England Journal of Medicine*.

[71] Battle RW, Davitt MA, Cooper SM (1996). Prevalence of pulmonary hypertension in limited and diffuse scleroderma. *Chest*.

[72] Hachulla E, Gressin V, Guillevin L (2005). Early detection of pulmonary arterial hypertension in systemic sclerosis: a French nationwide prospective multicenter study. *Arthritis and Rheumatism*.

[73] Williams MH, Das C, Handler CE (2006). Systemic sclerosis associated pulmonary hypertension: improved survival in the current era. *Heart*.

[74] Condliffe R, Kiely DG, Peacock AJ (2009). Connective tissue disease-associated pulmonary arterial hypertension in the modern treatment era. *American Journal of Respiratory and Critical Care Medicine*.

[75] Hachulla E, Carpentier P, Gressin V (2009). Risk factors for death and the 3-year survival of patients with systemic sclerosis: the French ItinérAIR-Sclérodermie study. *Rheumatology*.

[76] Hachulla E, Launay D, Yaici A (2010). Pulmonary arterial hypertension associated with systemic sclerosis in patients with functional class II dyspnoea: mild symptoms but severe outcome. *Rheumatology*.

[77] Chung L, Liu J, Parsons L (2010). Characterization of connective tissue disease-associated pulmonary arterial hypertension from REVEAL: identifying systemic sclerosis as a unique phenotype. *Chest*.

[78] Pisetsky DS, Gilkeson G, St Clair EW (1997). Systemic lupus erythematosus: diagnosis and treatment. *Medical Clinics of North America*.

[79] Asherson RA, Mackworth Young CG, Boey ML (1983). Pulmonary hypertension in systemic lupus erythematosus. *British Medical Journal*.

[80] Kasparian A, Floros A, Gialafos E (2007). Raynaud’s phenomenon is correlated with elevated systolic pulmonary arterial pressure in patients with systemic lupus erythematosus. *Lupus*.

[81] Pan TLT, Thumboo J, Boey ML (2000). Primary and secondary pulmonary hypertension in systemic lupus erythematosus. *Lupus*.

[82] Orens JB, Martinez FJ, Lynch JP (1994). Pleuropulmonary manifestations of systemic lupus erythematosus. *Rheumatic Disease Clinics of North America*.

[83] Fernandez-Alonso J, Zulueta T, Reyes-Ramirez JR, Castillo-Palma MJ, Sanchez-Roman J (1999). Pulmonary capillary hemangiomatosis as cause of pulmonary hypertension in a young woman with systemic lupus erythematosus. *Journal of Rheumatology*.

[84] Woolf D, Voigt MD, Jaskiewicz K, Kalla AA (1994). Pulmonary hypertension associated with non-cirrhotic portal hypertension in systemic lupus erythematosus. *Postgraduate Medical Journal*.

[85] De Clerck LS, Michielsen PP, Ramael MR (1991). Portal and pulmonary vessel thrombosis associated with systemic lupus erythematosus and anticardiolipin antibodies. *Journal of Rheumatology*.

[86] Hubscher O, Eimon A, Elsner B, Arana RM (1984). Fatal post-partum pulmonary vasculitis in systemic lupus erythematosus. *Clinical Rheumatology*.

[87] Haupt HM, Moore GW, Hutchins GM (1981). The lung in systemic lupus erythematosus. Analysis of the pathologic changes in 120 patients. *American Journal of Medicine*.

[88] Rubin LA, Geran A, Rose TH, Cohen H (1995). A fatal pulmonary complication of lupus in pregnancy. *Arthritis and Rheumatism*.

[89] Sasaki N, Kamataki A, Sawai T (2011). A histopathological study of pulmonary hypertension in connective tissue disease. *Allergology International*.

[90] Kamen DL, Strange C (2010). Pulmonary manifestations of systemic lupus erythematosus. *Clinics in Chest Medicine*.

[91] Sanchez O, Humbert M, Sitbon O, Simonneau G (1999). Treatment of pulmonary hypertension secondary to connective tissue diseases. *Thorax*.

[92] Haas C (2004). Pulmonary hypertension associated with systemic lupus erythematosus. *Bulletin de l'Academie Nationale de Medecine*.

[93] Yeh TT, Yang YH, Lin YT, Lu CS, Chiang BL (2007). Cardiopulmonary involvement in pediatric systemic lupus erythematosus: a twenty-year retrospective analysis. *Journal of Microbiology, Immunology and Infection*.

[94] Prabu A, Patel K, Yee CS (2009). Prevalence and risk factors for pulmonary arterial hypertension in patients with lupus. *Rheumatology*.

[95] Dawson JK, Goodson NG, Graham DR, Lynch MP (2000). Raised pulmonary artery pressures measured with Doppler echocardiography in rheumatoid arthritis patients. *Rheumatology*.

[96] Majithia V, Geraci SA (2007). Rheumatoid arthritis: diagnosis and management. *American Journal of Medicine*.

[97] Crestani B (2005). The respiratory system in connective tissue disorders. *Allergy*.

[98] Picchianti-Diamanti A, Germano V, Bizzi E, Laganà B, Migliore A (2011). Interstitial lung disease in rheumatoid arthritis in the era of biologics. *Pulmonary Medicine*.

[99] Fauchais AL, Martel C, Vidal E (2012). Epidemiology and physiopathogy of Sjögren's syndrome. *Revue du Praticien*.

[100] Hatron PY, Tillie-Leblond I, Launay D, Hachulla E, Fauchais AL, Wallaert B (2011). Pulmonary manifestations of Sjögren’s syndrome. *Presse Médicale*.

[101] Launay D, Hachulla E, Hatron PY, Jais X, Simonneau G, Humbert M (2007). Pulmonary arterial hypertension: a rare complication of primary Sjögren syndrome—report of 9 new cases and review of the literature. *Medicine*.

[102] Hunninghake GW, Costabel U, Ando M (1999). ATS/ERS/WASOG statement on sarcoidosis. *Sarcoidosis Vasculitis and Diffuse Lung Disease*.

[103] Tsangaris I, Orfanos S, Bouros D (2010). Pulmonary hypertension and lung diseases: a suggestion for revision of the clinical classification. *European Respiratory Journal*.

[104] Baughman RP, Teirstein AS, Judson MA (2001). Clinical characteristics of patients in a case control study of sarcoidosis. *American Journal of Respiratory and Critical Care Medicine*.

[105] Nunes H, Humbert M, Capron F (2006). Pulmonary hypertension associated with sarcoidosis: mechanisms, haemodynamics and prognosis. *Thorax*.

[106] Battesti JP, Georges R, Basset F, Saumon G (1978). Chronic cor pulmonale in pulmonary sarcoidosis. *Thorax*.

[107] Rizzato G, Pezzano A, Sala G (1983). Right heart impairment in sarcoidosis: haemodynamic and echocardiographic study. *European Journal of Respiratory Diseases*.

[108] Gluskowski J, Hawrylkiewicz I, Zych D (1984). Pulmonary haemodynamics at rest and during exercise in patients with sarcoidosis. *Respiration*.

[109] Rosen Y, Moon S, Huang CT (1977). Granulomatous pulmonary angiitis in sarcoidosis. *Archives of Pathology and Laboratory Medicine*.

[110] Takemura T, Matsui Y, Saiki S, Mikami R (1992). Pulmonary vascular involvement in sarcoidosis: a report of 40 autopsy cases. *Human Pathology*.

[111] Shorr AF, Helman DL, Davies DB, Nathan SD (2005). Pulmonary hypertension in advanced sarcoidosis: epidemiology and clinical characteristics. *European Respiratory Journal*.

[112] Portier F, Lerebours-Pigeonniere G, Thiberville L (1991). Sarcoidosis simulating pulmonary veno-occlusive disease. *Revue des Maladies Respiratoires*.

[113] Preston IR, Klinger JR, Landzberg MJ, Houtchens J, Nelson D, Hill NS (2001). Vasoresponsiveness of sarcoidosis-associated pulmonary hypertension. *Chest*.

[114] Salazar A, Mana J, Sala J, Landoni BR, Manresa F (1994). Combined portal and pulmonary hypertension in sarcoidosis. *Respiration*.

[115] Handa T, Nagai S, Miki S (2006). Incidence of pulmonary hypertension and its clinical relevance in patients with sarcoidosis. *Chest*.

[116] Bourbonnais JM, Samavati L (2008). Clinical predictors of pulmonary hypertension in sarcoidosis. *European Respiratory Journal*.

[117] Sulica R, Teirstein AS, Kakarla S, Nemani N, Behnegar A, Padilla ML (2005). Distinctive clinical, radiographic, and functional characteristics of patients with sarcoidosis-related pulmonary hypertension. *Chest*.

[118] Baughman RP, Engel PJ, Meyer CA, Barrett AB, Lower EE (2006). Pulmonary hypertension in sarcoidosis. *Sarcoidosis Vasculitis and Diffuse Lung Diseases*.

[119] American Thoracic Society and European Thoracic Society (2002). American Thoracic Society/European Respiratory Society International Multidisciplinary Consensus Classification of the Idiopathic Interstitial Pneumonias. *American Journal of Respiratory and Critical Care Medicine*.

[120] Flaherty KR, King TE, Raghu G (2004). Idiopathic interstitial pneumonia: what is the effect of a multidisciplinary approach to diagnosis?. *American Journal of Respiratory and Critical Care Medicine*.

[121] Cottin V, Nunes H, Brillet PY (2005). Combined pulmonary fibrosis and emphysema: a distinct underrecognised entity. *European Respiratory Journal*.

[122] Corte TJ, Wort SJ, Wells AU (2009). Pulmonary hypertension in idiopathic pulmonary fibrosis: a review. *Sarcoidosis Vasculitis and Diffuse Lung Diseases*.

[123] Kinnula VL, Fattman CL, Tan RJ, Oury TD (2005). Oxidative stress in pulmonary fibrosis: a possible role for redox modulatory therapy. *American Journal of Respiratory and Critical Care Medicine*.

[124] Nadrous HF, Pellikka PA, Krowka MJ (2005). Pulmonary hypertension in patients with idiopathic pulmonary fibrosis. *Chest*.

[125] Nathan SD, Noble PW, Tuder RM (2007). Idiopathic pulmonary fibrosis and pulmonary hypertension: connecting the dots. *American Journal of Respiratory and Critical Care Medicine*.

[126] Hamada K, Nagai S, Tanaka S (2007). Significance of pulmonary arterial pressure and diffusion capacity of the lung as prognosticator in patients with idiopathic pulmonary fibrosis. *Chest*.

[127] Ebina M, Shimizukawa M, Shibata N (2004). Heterogeneous increase in CD34-positive alveolar capillaries in idiopathic pulmonary fibrosis. *American Journal of Respiratory and Critical Care Medicine*.

[128] Cosgrove GP, Brown KK, Schiemann WP (2004). Pigment epithelium-derived factor in idiopathic pulmonary fibrosis: a role in aberrant angiogenesis. *American Journal of Respiratory and Critical Care Medicine*.

[129] Strieter RM, Starko KM, Enelow RI (2004). Effects of interferon-*γ* 1b on biomarker expression in patients with idiopathic pulmonary fibrosis. *American Journal of Respiratory and Critical Care Medicine*.

[130] Burdick MD, Murray LA, Keane MP (2005). CXCCL11 attenuates bleomycin-induced pulmonary fibrosis via inhibition of vascular remodeling. *American Journal of Respiratory and Critical Care Medicine*.

[131] Simler NR, Brenchley PE, Horrocks AW, Greaves SM, Hasleton PS, Egan JJ (2004). Angiogenic cytokines in patients with idiopathic interstitial pneumonia. *Thorax*.

[132] Renzoni EA, Walsh DA, Salmon M (2003). Interstitial vascularity in fibrosing alveolitis. *American Journal of Respiratory and Critical Care Medicine*.

[133] Gagermeier J, Dauber J, Yousem S, Gibson K, Kaminski N (2005). Abnormal vascular phenotypes in patients with idiopathic pulmonary fibrosis and secondary pulmonary hypertension. *Chest*.

[134] Charbeneau RP, Peters-Golden M (2005). Eicosanoids: mediators and therapeutic targets in fibrotic lung disease. *Clinical Science*.

[135] King TE, Behr J, Brown KK, du Bois RM, Raghu G (2006). Bosentan use in idiopathic pulmonary fibrosis (IPF): results of the placebo-controlled BUILD-1 study. *Proceedings of the American Thoracic Society*.

[136] Giaid A, Michel RP, Steward DJ, Sheppard M, Corrin B, Hamid Q (1993). Expression of endothelin-1 in lungs of patients with cryptogenic fibrosing alveolitis. *The Lancet*.

[137] Saleh D, Furukawa K, Tsao MS (1997). Elevated expression of endothelin-1 and endothelin-converting enzyme-1 in idiopathic pulmonary fibrosis: possible involvement of proinflammatory cytokines. *American Journal of Respiratory Cell and Molecular Biology*.

[138] Trakada G, Spiropoulos K (2001). Arterial endothelin-1 in interstitial lung disease patients with pulmonary hypertension. *Monaldi Archives for Chest Disease*.

[139] Richter A, Yeager ME, Zaiman A, Cool CD, Voelkel NF, Tuder RM (2004). Impaired transforming growth factor-*β* signaling in idiopathic pulmonary arterial hypertension. *American Journal of Respiratory and Critical Care Medicine*.

[140] Nathan SD, Shlobin OA, Ahmad S (2008). Serial development of pulmonary hypertension in patients with idiopathic pulmonary fibrosis. *Respiration*.

[141] Fang A, Studer S, Kawut SM (2011). Elevated pulmonary artery pressure is a risk factor for primary graft dysfunction following lung transplantation for idiopathic pulmonary fibrosis. *Chest*.

[142] Yang S, Johnson C, Hoffman K, Mulligan M, Spada C, Raghu G (2006). Pulmonary arterial hypertension in patients with idiopathic pulmonary fibrosis when listed for lung transplantation (LT) and at LT. *Proceedings of the American Thoracic Society*.

[143] Kitaichi M, Nishimura K, Itoh H, Izumi T (1995). Pulmonary lymphangioleiomyomatosis: a report of 46 patients including a clinicopathologic study of prognostic factors. *American Journal of Respiratory and Critical Care Medicine*.

[144] Sullivan EJ (1998). Lymphangioleiomyomatosis. *Chest*.

[145] Chu SC, Horiba K, Usuki J (1999). Comprehensive evaluation of 35 patients with lymphangioleiomyomatosis. *Chest*.

[146] Urban TJ, Lazor R, Lacronique J (1999). Pulmonary lymphangioleiomyomatosis: a study of 69 patients. *Medicine*.

[147] Johnson S (1999). Lymphangioleiomyomatosis: clinical features, management and basic mechanisms. *Thorax*.

[148] Ryu JH, Moss J, Beck GJ (2006). The NHLBI lymphangioleiomyomatosis registry: characteristics of 230 patients at enrollment. *American Journal of Respiratory and Critical Care Medicine*.

[149] Taveira-DaSilva AM, Hedin C, Stylianou MP (2001). Reversible airflow obstruction, proliferation of abnormal smooth muscle cells, and impairment of gas exchange as predictors of outcome in lymphangioleiomyomatosis. *American Journal of Respiratory and Critical Care Medicine*.

[150] Taveira-DaSilva AM, Stylianou MP, Hedin CJ (2003). Maximal oxygen uptake and severity of disease in lymphangioleiomyomatosis. *American Journal of Respiratory and Critical Care Medicine*.

[151] Taveira-DaSilva AM, Hathaway OM, Sachdev V, Shizukuda Y, Birdsall CW, Moss J (2007). Pulmonary artery pressure in lymphangioleiomyomatosis: an echocardiographic study. *Chest*.

[152] Ansótegui Barrera E, Mancheño Franch N, Peñalver Cuesta JC, Vera-Sempere F, Padilla Alarcón J (2012). Sporadic lymphangioleiomyomatosis and pulmonary hypertension. Clinical and pathologic study in patients undergoing lung transplantation. *Medicina Clinica*.

[153] Cottin V V, Harari S, Humbert M, Mal H, Dorfmüller P, Jais X (2012). Pulmonary hypertension in lymphangioleiomyomatosis: characteristics in 20 patients. *European Respiratory Journal*.

[154] Sekhon HS, Wright JL, Churg A (1994). Cigarette smoke causes rapid cell proliferation in small airways and associated pulmonary arteries. *American Journal of Physiology*.

[155] Hale KA, Ewing SL, Gosnell BA, Niewoehner DE (1984). Lung disease in long-term cigarette smokers with and without chronic air-flow obstruction. *American Review of Respiratory Disease*.

[156] Zhu ZG, Li HH, Zhang BR (1997). Expression of endothelin-1 and constitutional nitric oxide synthase messenger RNA in saphenous vein endothelial cells exposed to arterial flow shear stress. *Annals of Thoracic Surgery*.

[157] Stamler JS, Loh E, Roddy MA, Currie KE, Creager MA (1994). Nitric oxide regulates basal systemic and pulmonary vascular resistance in healthy humans. *Circulation*.

[158] Barberà JA, Peinado VI, Santos S, Ramirez J, Roca J, Rodriguez-Roisin R (2001). Reduced expression of endothelial nitric oxide synthase in pulmonary arteries of smokers. *American Journal of Respiratory and Critical Care Medicine*.

[159] Barberà JA, Peinado VI, Santos S (2003). Pulmonary hypertension in chronic obstructive pulmonary disease. *European Respiratory Journal*.

[160] Dinh-Xuan AT, Higenbottam TW, Clelland CA (1991). Impairment of endothelium-dependent pulmonary-artery relaxation in chronic obstructive lung disease. *The New England Journal of Medicine*.

[161] Yildiz P, Oflaz H, Cine N, Erginel-Ünaltuna N, Erzengin F, Yilmaz V (2003). Gene polymorphisms of endothelial nitric oxide synthase enzyme associated with pulmonary hypertension in patients with COPD. *Respiratory Medicine*.

[162] Joppa P, Petrasova D, Stancak B, Tkacova R (2006). Systemic inflammation in patients with COPD and pulmonary hypertension. *Chest*.

[163] Chaouat A, Savale L, Chouaid C (2009). Role for interleukin-6 in COPD-related pulmonary hypertension. *Chest*.

[164] McFarlane PA, Gardaz JP, Sykes MK (1984). CO_2_ and mechanical factors reduce blood flow in a collapsed lung lobe. *Journal of Applied Physiology Respiratory Environmental and Exercise Physiology*.

[165] Eddahibi S, Chaouat A, Morrell N (2003). Polymorphism of the serotonin transporter gene and pulmonary hypertension in chronic obstructive pulmonary disease. *Circulation*.

[166] Peinado VI, Barbera JA, Ramirez J (1998). Endothelial dysfunction in pulmonary arteries of patients with mild COPD. *American Journal of Physiology*.

[167] McGrath RL, Weil JV (1978). Adverse effects of normovolemic polycythemia and hypoxia on hemodynamics in the dog. *Circulation Research*.

[168] Hasegawa J, Wagner KF, Karp D (2004). Altered pulmonary vascular reactivity in mice with excessive erythrocytosis. *American Journal of Respiratory and Critical Care Medicine*.

[169] Scharf SM, Iqbal M, Keller C, Criner G, Lee S, Fessler HE (2002). Hemodynamic characterization of patients with severe emphysema. *American Journal of Respiratory and Critical Care Medicine*.

[170] Arcasoy SM, Christie JD, Ferrari VA (2003). Echocardiographic assessment of pulmonary hypertension in patients with advanced lung disease. *American Journal of Respiratory and Critical Care Medicine*.

[171] Fayngersh V, Drakopanagiotakis F, Dennis McCool F, Klinger JR (2011). Pulmonary hypertension in a stable community-based COPD population. *Lung*.

[172] Fisher MR, Forfia PR, Chamera E (2009). Accuracy of doppler echocardiography in the hemodynamic assessment of pulmonary hypertension. *American Journal of Respiratory and Critical Care Medicine*.

[173] Chaouat A, Naeije R, Weitzenblum E (2008). Pulmonary hypertension in COPD. *European Respiratory Journal*.

[174] Oswald-Mammosser M, Weitzenblum E, Quoix E (1995). Prognostic factors in COPD patients receiving long-term oxygen therapy: importance of pulmonary artery pressure. *Chest*.

